# Navigating Calcified Challenges: Guided Endodontic Treatment of a Maxillary Central Incisor

**DOI:** 10.1002/ccr3.70627

**Published:** 2025-07-21

**Authors:** Saide Nabavi, Sara Navabi, Iman Shiezadeh, SeyedehZahra JamaliMotlagh

**Affiliations:** ^1^ Endodontic Department, Faculty of Dentistry Mashhad University of Medical Sciences Mashhad Iran; ^2^ Student Research Committee, Faculty of Dentistry Mashhad University of Medical Sciences Mashhad Iran

**Keywords:** 3D‐printed guide, calcified canal, guided endodontics, minimally invasive dentistry, pulp canal obliteration

## Abstract

This case report details the guided endodontic treatment of a calcified maxillary central incisor in a 47‐year‐old patient with a history of trauma. Traditional methods pose challenges in managing pulp canal obliteration (PCO) due to the risk of perforation and excessive dentin removal. Guided endodontics, integrating cone‐beam computed tomography (CBCT) and intraoral scanning, was employed to design a 3D‐printed guide, allowing precise, minimally invasive access to the obliterated canal. A customized guide facilitated accurate drilling with minimal tooth structure loss. The canal was successfully negotiated, cleaned, shaped, and obturated. Posttreatment, the tooth underwent internal bleaching to address discoloration. At the 18‐month follow‐up, the tooth was asymptomatic, functional, and showed complete periapical healing. This report highlights the efficacy of guided endodontics in managing calcified canals, emphasizing its precision, reduced procedural risks, and preservation of dental structure. While challenges such as guide accuracy and workflow complexity remain, advances in digital technology continue to enhance clinical outcomes in endodontics.

**Summary**

- Guided endodontic treatment using 3D‐printed templates offers a precise, minimally invasive approach for managing calcified canals.
- This technique significantly reduces the risk of perforation and excessive dentin removal while preserving tooth structure, providing a predictable treatment option for challenging cases of pulp canal obliteration.

## 1 Introduction

The field of endodontics has long been challenged by the complexities of treating calcified root canals. Calcification often results from trauma or aging, leading to pulp canal obliteration (PCO) that complicates conventional root canal therapy (RCT). Traditional methods of accessing these calcified canals can be invasive and carry a significant risk of iatrogenic damage, including perforations and excessive loss of tooth structure [1]. However, the advent of guided endodontics has revolutionized the approach to such challenging cases.

Guided endodontics (GE) integrates cone‐beam computed tomography (CBCT) with digital intraoral scans to produce precise 3D models of the tooth and its surrounding structures. This approach allows for the design and fabrication of custom endodontic guides, which facilitate minimally invasive access to the root canal system [2]. These guides ensure accurate drilling paths, reducing the risk of deviations and perforations, and preserving healthy tooth structure [3]. The technique has been particularly beneficial in managing calcified canals, where traditional methods might fail to locate the canal or cause undue damage [4].

Recently, several reports have highlighted the efficacy of guided endodontics in treating calcified incisors.

The process of GE begins with acquiring high‐resolution CBCT images, which provide detailed insights into the tooth's internal anatomy. These images are combined with digital surface scans to create a comprehensive 3D model. Software such as Blue‐Sky Bio or Implant Viewer is used to plan the access path, ensuring that the guide directs the drill to the precise location of the canal [1, 3]. This digital planning stage is crucial, as it allows for the customization of the guide to the specific anatomy of each tooth, accommodating variations such as canal curvature or calcification extent [2].

The implementation of edno guides in clinical practice not only enhances the precision of RCT but also significantly reduces chair time and improves patient outcomes. The technique's minimally invasive nature helps preserve the structure of the tooth, which is critical for maintaining its long‐term function and esthetics [4]. Moreover, guided endodontics can be performed without the need for an operating microscope, making it accessible to a broader range of practitioners [5].

Despite its advantages, the accuracy of the guides depends on the quality of the CBCT images and the precision of the 3D printing process. In some cases, inaccuracies in the guide design or placement can lead to complications such as root perforation [5]. Therefore, careful planning and execution are essential to maximize the benefits of this technology.

Guided endodontics represents a significant advancement in the treatment of calcified root canals, offering a more predictable and conservative approach compared to traditional methods. This case report will demonstrate the application and effectiveness of guided endodontic treatment in managing a calcified maxillary central incisor.

## 2 Case History/Examination

**FIGURE 1:**
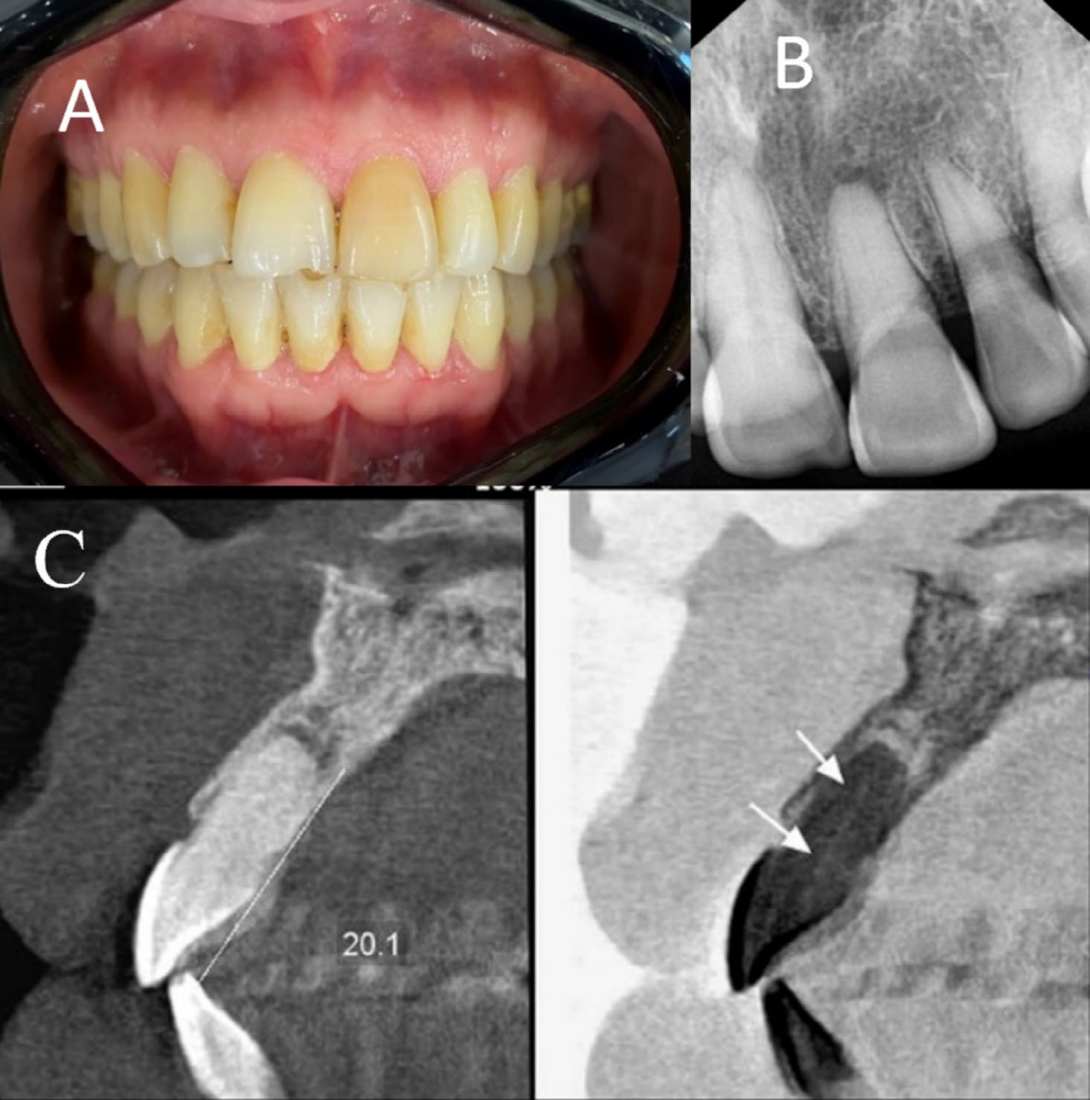
(A) Clinical view shows discoloration in tooth 8#, (B) periapical radiograph shows pulp canal calcification, and (C) CBCT image, the sagittal view shows pulp canal obliteration in the coronal thirds.

A 47‐year‐old female patient with no significant medical history was referred to a private dental clinic for root canal treatment of the left maxillary incisor (tooth 8#) with a chief complaint of discoloration and chewing pain. Clinical examination revealed pain upon percussion but no evidence of a sinus tract (Figure 1A). Radiographic analysis showed obliteration of the root canal system in the affected tooth, accompanied by a periapical lesion (Figure 1B). Based on clinical and radiographic findings, a diagnosis of pulp necrosis was confirmed. Notably, the patient reported a history of trauma to the tooth during childhood.

## 3 Differential Diagnosis, Investigations and Treatment

**FIGURE 2:**
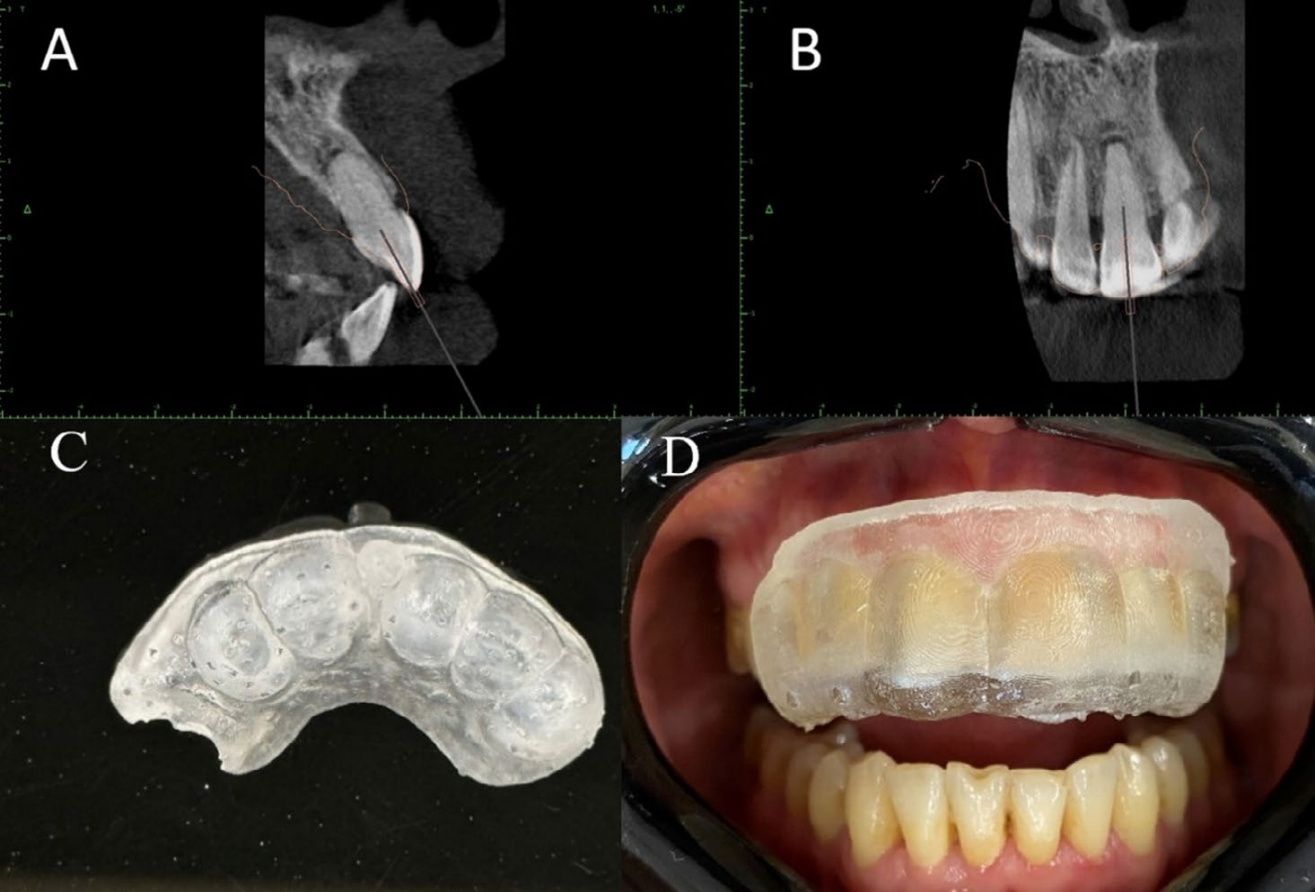
(A, B) Axial and frontal view of the virtual planning of the guided endodontic template in CBCT image, (C) The printed resin template, (D) evaluating the stability of the template in the mouth.

To further assess the extent of PCO and determine the root canal position in different planes, a cone beam computed tomography (CBCT) scan was requested. The CBCT imaging (ProMax 3D MAX, Planmeca OY, Helsinki, Finland) indicated a calcified root canal in the coronal third of the root (Figure 1C). To prevent iatrogenic complications during treatment, such as perforation or deviation from the main canal path, a 3D‐printed guide was designed to assist in navigating the procedure. An intraoral scan was performed using the Primescan scanner (Dentsply Sirona, Germany) to meticulously plan and design the guide. The digital imaging and communications in medicine (DICOM) files from the CBCT and standard tessellation language (STL) files from the intraoral scan were imported into a Blue‐Sky Plan Software (Blue Sky Bio LLC, Glenview, Illinois, USA), which is typically used for designing endodontic guides. During the planning phase, a specific drill path was created for a size 1 Munce Discovery bur (CJM Engineering, Santa Barbara, CA, USA) by integrating the bur dimensions into the template. After completing the design of the endo guide (Figure 2A,B) a 3D guide (Figure 2C) was printed using a Sonic 4 K 3D Printer (Phrozen Technology, Taiwan).

**FIGURE 3:**
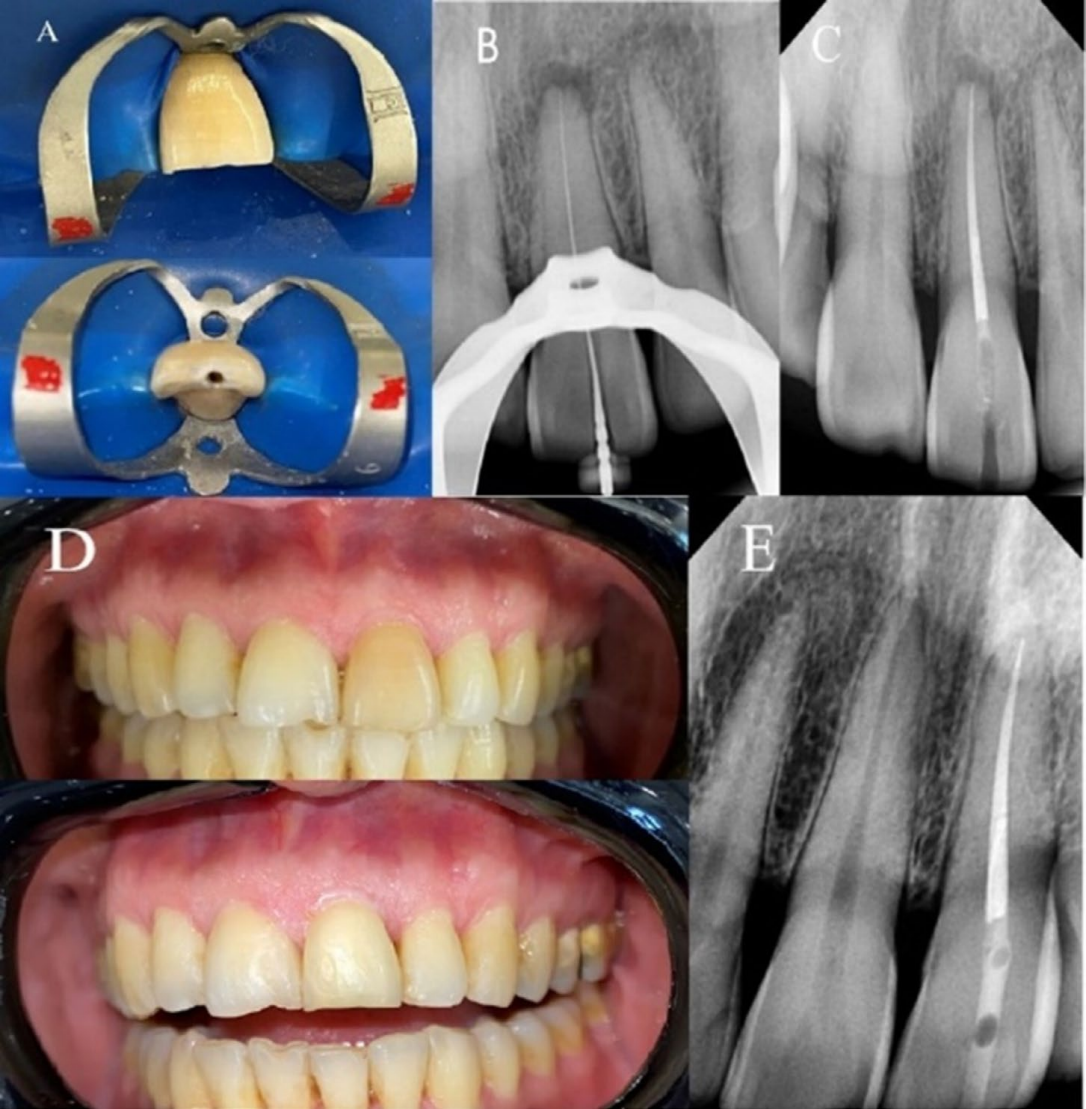
(A) initial file and checking SLA, (B) master cone fit checking, (C) conservative access cavity and obturation with glass ionomer and light‐cured temporary filling, (D) clinical view of post endo bleaching process, and (E) 18‐month follow‐up.

In the clinical session prior to treatment initiation, the guide was evaluated, focusing on both the fit and stability of the guide in the region (Figure 2D). A pilot drill hole was created in the tooth using a diamond bur to facilitate the drill placement through the guide. The access cavity was prepared using the Munce Discovery bur (CJM Engineering, Santa Barbara, CA, USA) while maintaining the integrity of the tooth structure as much as possible. The template was periodically removed to check the exposure using a C pilot 10 file (VDW Endodontic Synergy, Munich, Germany). With minimal tooth structure removal and maximum preservation of dental structure, the canal path was located using an endo guide (Figure 3A). Additionally, in the buccal view of the tooth, despite using only a single endo guide, no changes were made and no changes obvious in buccal view of the tooth (Figure 3A). Eventually, the canal was accessed, and patency was confirmed (Figure 3B).

During the procedure, the canal was irrigated with 25 mL of 5% sodium hypochlorite (Cerkamed, Stalowa Wola, Poland). The canal was shaped up to size 25 with rotary files (Hero 642, Micro Mega, France), followed by a final rinse with 3 mL of 17% EDTA (Cerkamed, Stalowa Wola, Poland). The canal was then dried with paper points (Meta Dental Co., Cheongju City, Korea) and obturated using the warm vertical condensation technique with AH Plus Jet sealer (Dentsply Maillefer, Ballaigues, Switzerland). For the bleaching process, the tooth was isolated using a rubber dam, and the gingiva was protected with a barrier cream. A 2 mm layer of glass ionomer was used to seal the orifice. A 35% hydrogen peroxide gel (Opalescent Endo; Jordan, UT, Ultradent, USA) was applied to the pulp chamber, and the cavity was sealed with Cavit (3 M ESPE, St. Paul, USA) (Figure 3C). The bleaching gel was replaced every 3–4 days until the desired tooth color was achieved.

## 4 Conclusion and Results (Outcome and Follow‐Up)

After 4 days, the color correction was satisfactory (Figure 3D), the bleaching gel was removed, and the cavity was temporarily sealed with Cavit. To ensure optimal bonding, the patient was referred to the restorative department 2 weeks after the bleaching procedure [6]. At the 18‐month follow‐up, the tooth remained asymptomatic, and the lesion had completely healed (Figure 3E) The reason for the different angle in the follow‐up image was that, due to a lack of access to the patient, they were asked to obtain and send a follow‐up radiograph from their city of residence.

Written informed consent was obtained from the patient, and she expressed her agreement to have her case reported for scientific purposes.

## 5 Discussion

The AAE considers PCO to be a highly challenging endodontic case that should be considered for referral [7]. Reports indicate a poor prognosis for root canal treatment in teeth where instrumentation is unsuccessful [8]. Although radiographs of calcified teeth may show no pulp canal space, histological examination reveals that most of these teeth still contain pulp tissue [6]. As it was confirmed in our case.

The American Association of Endodontists and the American Academy of Oral and Maxillofacial Radiography (AAE/AAOMR) advise using CBCT imaging in endodontics to identify calcified root canals [9].

For instance, Freire et al. [3] demonstrated the successful use of a 3D‐printed guide to access a calcified central incisor, resulting in complete healing of periapical tissues after 2 years. Similarly, Hegde et al. [1] reported on the management of PCO in a maxillary central incisor using guided endodontic therapy, emphasizing the technique's ability to locate canals with minimal removal of dentine.

The successful management of a calcified maxillary central incisor in our case report underscores the evolving role of guided endodontics as a valuable tool in navigating the complexities of PCO. This approach is increasingly recognized for its precision and ability to minimize iatrogenic damage, a significant advantage over traditional methods. Our findings are consistent with those of Ali and Arslan, who demonstrated the efficacy of guided endodontics in accessing complex root canal systems, including those with dens invaginatus, by significantly reducing chair time and the risk of tissue damage [10].

In previous centers, conventional approaches such as apicoectomy and indirect veneer restorations were proposed to manage the patient's calcified maxillary central incisor with PCO. These options inherently involve significant removal of tooth structure and are associated with greater risks of iatrogenic complications. However, in our case, the treatment was accomplished using a minimally invasive guided endodontic technique. By utilizing CBCT and intraoral scanning to design a 3D‐printed guide, we were able to precisely locate and access the obliterated canal while preserving maximum tooth structure. This approach not only avoided the need for invasive surgical procedures but also optimized the structural integrity and long‐term prognosis of the tooth.

The integration of CBCT and intraoral scanning to design a 3D‐printed guide is a technique which is utilized in our case [2]. The personalized nature of guided endodontics allows for a tailored approach to each patient's unique dental anatomy, particularly in cases of calcification. This customization is crucial in ensuring a minimally invasive approach, preserving as much tooth structure as possible.

Microscopic visualization reduces the risk of iatrogenic errors such as perforation and excessive structure removal, which are identified as common complications in calcified cases [11]. Unfortunately, despite our recommendation, the patient declined to have the final restoration placed under microscopic magnification by the same practitioner who performed the endodontic treatment and instead sought restorative care elsewhere. The visible voids present in the composite restoration (Figure 3E) can be attributed to this decision, as optimal adaptation of composite materials in anterior teeth with minimally invasive access preparations benefits significantly from microscopic visualization. The integration of microscopes with guided endodontic procedures represents a synergistic approach that maximizes technological advantages while respecting biological principles.

Contrary to previous methodologies described in the literature, such as the study by Zargar et al. [6], our access cavity preparation was exceptionally conservative and approached from the incisal edge. This minimally invasive technique preserved substantial coronal tooth structure while still providing adequate access to the canal system. The incisal approach required extraordinary precision in alignment and execution, effectively doubling the technical demands of the procedure. This conservative design not only maximizes the structural integrity of the tooth but also optimizes the long‐term restorative prognosis by maintaining natural esthetics and functional load‐bearing capacity. The successful implementation of this technique demonstrates the evolving nature of guided endodontic procedures toward increasingly conservative approaches that prioritize structural preservation without compromising treatment efficacy.

Our use of a 3D‐printed guide facilitated the precise location and access of the obliterated canal, a technique supported by Costamagna et al. [12], who emphasized the importance of preoperative planning through CBCT and 3D modeling in managing complex tooth anatomy. Similarly, Fonseca Tavares et al. [13] illustrated the reliability of guided endodontics in treating calcified molars, further supporting the versatility of this technique across different tooth types.

Despite its advantages, guided endodontics is not without limitations. As noted, inaccuracies in guide placement or errors during the digital workflow can lead to complications such as root perforation [5]. This underscores the need for meticulous planning and execution, as well as the potential requirement for additional training for clinicians new to this technology.

Additionally, the study by Kamburoğlu et al. [14] raised concerns about the accuracy of CBCT‐guided 3D‐printed guides across different materials and layer thicknesses. Ensuring the precision of guides remains a critical factor for successful outcomes.

On the other hand, the innovative use of a sleeveless, open‐frame titanium guide, as introduced by Fornara et al. [4], presents a promising advancement in guided endodontics, offering cost savings and reduced chairside time compared to traditional templates. This approach holds potential for enhancing the efficiency and quality of endodontic procedures, particularly in cases of PCO.

## 6 Conclusion

In conclusion, our case report adds to the growing body of evidence supporting the use of guided endodontics as a safe, efficient, and minimally invasive approach for managing calcified canals. While further studies are needed to explore its broader applicability and to refine techniques, the current findings suggest that guided endodontics can significantly enhance treatment outcomes, reduce procedural risks, and preserve dental structures. As digital technologies continue to evolve, they promise to further revolutionize the field of endodontics, offering new solutions for previously challenging clinical scenarios.

## Author Contributions

**Saide Nabavi:** conceptualization, methodology, project administration, validation, writing – review and editing. **Sara Navabi:** conceptualization, data curation, investigation, methodology, project administration, writing – review and editing. **Iman Shiezadeh:** data curation, methodology, project administration, supervision, writing – original draft, writing – review and editing. **SeyedehZahra JamaliMotlagh:** project administration, software, writing – original draft, writing – review and editing.

## Ethics Statement

For the clinical case, the local ethics committee considers that the patient's consent is sufficient.

## Consent

Written informed consent was obtained from the patient to publish this report in accordance with the journal's patient consent policy, which can be provided to the publisher if requested.

## Conflicts of Interest

The authors declare no conflicts of interest.

## Data Availability

The clinical pictures and radiographs data that support the findings of this study are included in the article.
